# Characterization of the Melanoma miRNAome by Deep Sequencing

**DOI:** 10.1371/journal.pone.0009685

**Published:** 2010-03-12

**Authors:** Mitchell S. Stark, Sonika Tyagi, Derek J. Nancarrow, Glen M. Boyle, Anthony L. Cook, David C. Whiteman, Peter G. Parsons, Christopher Schmidt, Richard A. Sturm, Nicholas K. Hayward

**Affiliations:** 1 Oncogenomics Laboratory, Queensland Institute of Medical Research, Herston, Brisbane, Queensland, Australia; 2 Drug discovery Laboratory, Queensland Institute of Medical Research, Herston, Brisbane, Queensland, Australia; 3 Cancer Control Group, Queensland Institute of Medical Research, Herston, Brisbane, Queensland, Australia; 4 Melanogenix Group, Institute for Molecular Bioscience, University of Queensland, St Lucia, Queensland, Australia; 5 Cancer Immunotherapy Group, Queensland Institute of Medical Research, Herston, Brisbane, Queensland, Australia; The University of Queensland, Australia

## Abstract

**Background:**

MicroRNAs (miRNAs) are 18–23 nucleotide non-coding RNAs that regulate gene expression in a sequence specific manner. Little is known about the repertoire and function of miRNAs in melanoma or the melanocytic lineage. We therefore undertook a comprehensive analysis of the miRNAome in a diverse range of pigment cells including: melanoblasts, melanocytes, congenital nevocytes, acral, mucosal, cutaneous and uveal melanoma cells.

**Methodology/Principal Findings:**

We sequenced 12 small RNA libraries using Illumina's Genome Analyzer II platform. This massively parallel sequencing approach of a diverse set of melanoma and pigment cell libraries revealed a total of 539 known mature and mature-star sequences, along with the prediction of 279 novel miRNA candidates, of which 109 were common to 2 or more libraries and 3 were present in all libraries.

**Conclusions/Significance:**

Some of the novel candidate miRNAs may be specific to the melanocytic lineage and as such could be used as biomarkers to assist in the early detection of distant metastases by measuring the circulating levels in blood. Follow up studies of the functional roles of these pigment cell miRNAs and the identification of the targets should shed further light on the development and progression of melanoma.

## Introduction

Small (18–23 nucleotide) non-coding RNAs called microRNAs (miRNAs) have been shown to regulate gene expression in a sequence specific manner. Originally this was thought to be accomplished solely through binding to the 3′ UTR of target mRNAs and either targeting the transcripts for degradation or blocking translation of the encoded protein, however, it is now evident that miRNAs can also stimulate translation [1] and regulate transcription through binding to gene promoters [2]. MiRNAs are thus central regulators of gene expression and can act both in a positive and negative way to control protein levels in the cell. MiRNA expression is generally down-regulated in cancer [3] and this appears to be due to defects in the processing of the immature primary miRNA transcripts (pri-miRNAs) to precursor miRNA transcripts (pre-miRNAs) by the enzyme Drosha [4]. The pre-miRNA stem-loop has the possibility of producing two mature miRNAs, one from the plus strand and one from the minus strand (miRNA*). Following cleavage by Drosha, pre-miRNAs are exported from the nucleus into the cytoplasm by Exportin 5 where these hairpin precursors are cleaved by Dicer into small, imperfect dsRNA duplexes (miRNA: miRNA^*^) that contains both the mature miRNA strand and its complementary strand (miRNA^*^) (reviewed in [5]). In most cases the miRNA* sequence is degraded leaving the mature sequence to be incorporated into the RISC complex and target mRNAs in a sequence-specific manner through the RNA interference pathway [5]. In cancer, particular miRNAs have been shown to be oncogenic [6], [7], [8], or conversely, tumor suppressive [9], [10].

Given their role in modulating gene expression it is not surprising that miRNAs have been shown to be intimately involved in regulating a wide variety of biological processes, including proliferation, apoptosis, cell-cycle control, and differentiation. In particular, miR-221 and miR-222 have been reported to regulate melanoma progression through p27 [11]. One of the most oncogenic miRNAs, miR-21 [12], has been shown to be involved in invasion and metastasis in many cancer types by its action on numerous genes involved in extracellular matrix modification, in particular PTEN (reviewed in [13]), a tumor suppressor gene involved in melanoma etiology [14].

Little is known about the repertoire and function of miRNAs in melanoma. A handful of studies have assessed the levels of subsets of miRNAs through microarray expression profiling [15], [16] or have assessed the mechanisms of action of select miRNAs in this tumor type [11], [17], [18], [19].

A greater understanding of the roles of individual miRNAs in melanoma development requires comprehensive analysis of the full complement of such molecules and their relative abundance. We thus set out to extend the repertoire of miRNAs in a diverse range of normal and malignant pigment cells through deep sequencing. We constructed 12 small RNA libraries from cell cultures of melanoblasts, melanocytes and nevocytes, as well as acral, mucosal, cutaneous and uveal melanomas. Using a series of bioinformatics tools we quantified known mature miRNA sequences and predicted novel miRNA candidates.

## Results

### Overview

**Figure 1:**
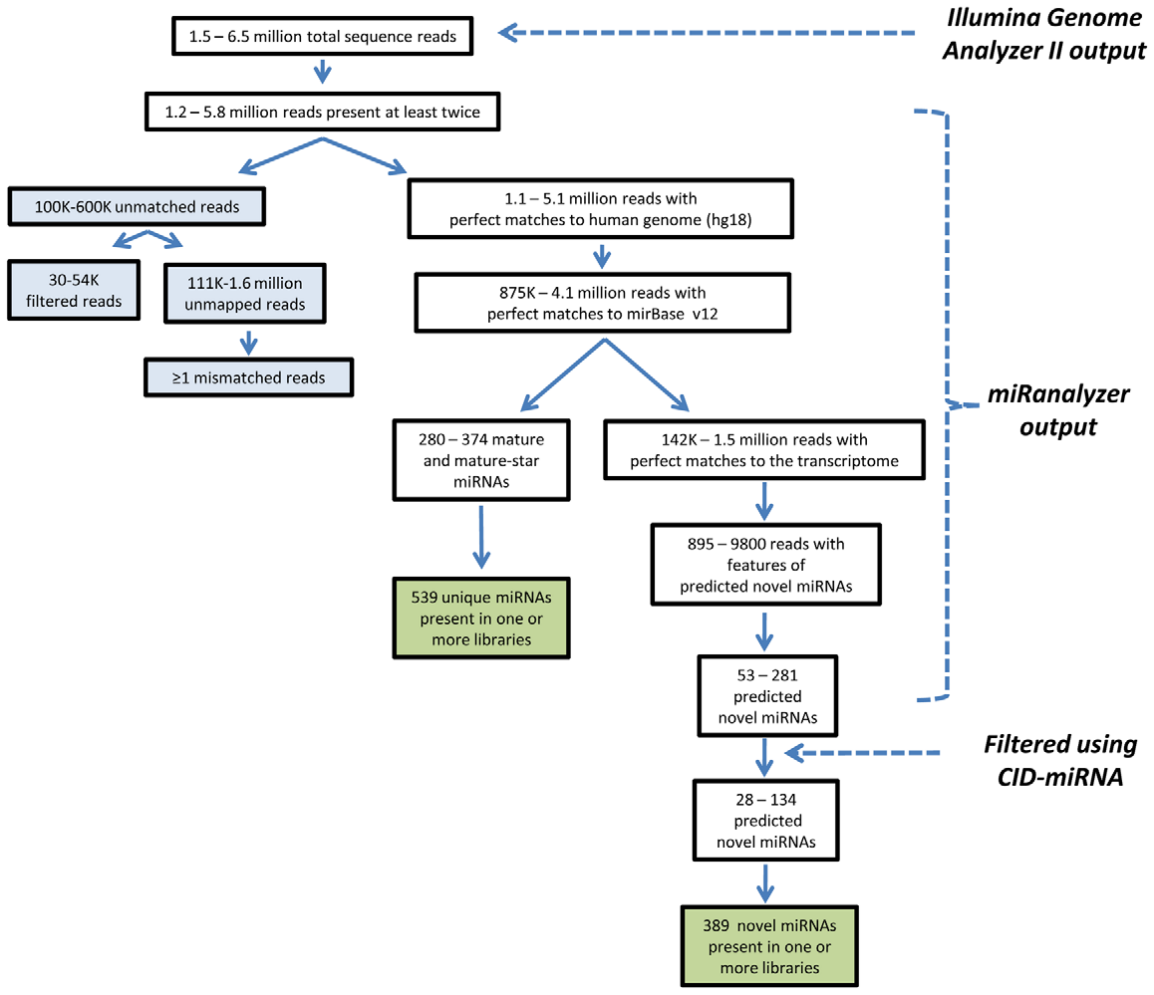
Flowchart representing the steps involved to annotate known microRNAs and the discovery of predicted candidate microRNAs using miRanalyzer [20] and CID-miRNA [21]. White boxes represent the range of numbers for an individual library. Green boxes represent the total number of unique miRNAs across all libraries. Blue boxes represent unmatched reads which were not considered further.

Each library was sequenced independently. Figure 1 shows a flowchart representing the steps involved to annotate known microRNAs and the discovery of the predicted candidate microRNAs using miRanalyzer [20] and CID-miRNA [21]. Briefly, between 1.5–6.5 million total sequence reads were obtained for a given library and this ranged from 1.2–5.8 million reads whose sequences were present at least twice. The number of perfect matches for these reads that aligned to the human genome (hg18) ranged from 1.1–5.1 million. Using miRanalyzer, all of the unique sequence counts were aligned to the known pre-miRNA sequences (miRBase v12, September 2008). These reads ranged from 875K–4.1 million which generated at total of 539 unique known miRNAs present in one or more libraries. To identify novel candidate miRNAs the miRanalyzer program was used to remove the known miRNAs from the pool, with the remainder then aligned to the hg18 transcriptome (142K–1.5 million reads) in order to filter out any potentially degraded mRNA sequences. These were then also removed from the pool. All sequence reads that passed this filtering process were then mapped to the human genome with 895–9800 reads, per library with features of predicted novel miRNAs. For each of these candidates the surrounding sequence was assessed to determine if it was able to form a hairpin structure using the Vienna RNAfold package [22], indicative of a pre-miRNA. Sequences that met this criterion were deemed to be novel candidate miRNAs (combined total of 722 predicted candidates across all libraries) These predicted novel miRNAs were filtered further using CID-miRNA [21] (for details see section “***Novel miRNAs***” below). This reduced the list to 279 novel candidate miRNAs which have a high likelihood of being bona fide miRNAs. Identical reads were tallied to determine their fraction amongst all unique sequence tags for each library. Both hg18-matched tags and the total number of tags varied considerably between libraries, as shown in the summary statistics (a detailed description of which can be found in Hackenberg *et al.* [20]) for each small RNA library presented in Tables S1A-S1L. All counts and predictions were generated using the miRanalyzer web server tool [20].

The melanoblast library (QF1160MB) had the highest number of unique sequence tags (77,261) and the melanocyte library (MELB) had the fewest (17,809) (Tables S1A and S1B respectively).

### Known mature and mature-star miRNAs

There are 847 human mature and mature-star miRNAs annotated in release 12.0 of miRBase (Sept 2008) [23]. The relative proportion of each of these was determined for each library (Table S1). A total of 539 mature and mature-star sequences (Table S2) were found across the 12 small RNA libraries. The total number in each library varied from 280 (D10) up to 374 (D20), with 180 sequences being common to all libraries (Table S3).

Hsa-let7f had the highest expression in all libraries, with the exception of MM466, where hsa-miR-29a was the highest. Table 1 shows the top 10 most abundant miRNAs in each library, which overall are represented by a non-redundant list of 23 miRNAs. A total of 96 miRNAs were found to be present in only a single library (Table S4).

**Table 1:** Rank order of the top 10 most common miRNAs in each library.

miRNA name	QF1160MB	MELB	MM653	D20	MM386	MM426	MM466	MM603	MM472	D10	D11	MEL202
hsa-let-7a	2	2	1	2	1	2	3	2	2	2	2	2
hsa-let-7b	5	3	2	3	5	3		4	3	4	5	5
hsa-let-7d		9						8		6	7	7
hsa-let-7e	6	4	8									9
hsa-let-7f	1	1	3	1	3	1	7	1	1	1	1	1
hsa-let-7g		10				10		7			4	8
hsa-let-7i	3	5	10	6	6				9	8	6	
hsa-miR-103	10			8	7	6	2	3	6		3	6
hsa-miR-140-3p									4	9	9	10
hsa-miR-146a						8						
hsa-miR-181a					10							
hsa-miR-185								10				
hsa-miR-21			6	5	4	4	8	9	5			
hsa-miR-211											10	
hsa-miR-221			4	7	9							
hsa-miR-222			9									
hsa-miR-25						9	10			10	8	
hsa-miR-29a	9	6	7	4	2	5	1	5	7	3		
hsa-miR-320a	4	8	5	9					8	7		3
hsa-miR-378	7	7		10		7	4			5		4
hsa-miR-423-5p	8				8		5		10			
hsa-miR-886-5p							6					
hsa-miR-92a							9	6				

The relative ratios of miRNA to miRNA* sequence reads ranged from 0.03∶1 to 242,000∶1 (Table S5). A total of 23 miRNAs were found to have roughly equivalent (0.75–2.2 relative ratio) counts generated from each arm of the pre-miRNA in at least one of the libraries (Table S5). However, 21 mature-star miRNAs were actually more abundant (0.01–0.74 relative ratio) than the corresponding mature sequences (Table S5). The remainder of the mature/mature-star miRNA ratios showed that the mature sequence was more highly expressed in the majority of the libraries (Table S5).

**Figure 2:**
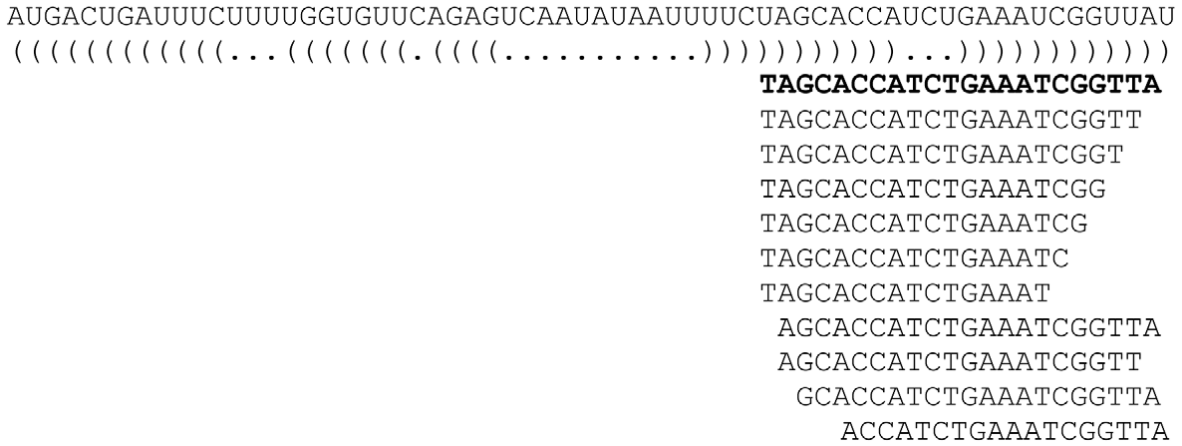
Example of a mature miRNA (hsa-mir-29a) showing variations in the 5′ and 3′ ends. The optimal secondary structure is represented in dot-bracket notation (where brackets and full-stops represent complementary and non-complementary nucleotides respectively) with the published mature miRNA bolded.

We observed heterogeneity in the start and end positions of the sequence tags. In all libraries, we noted a high proportion of sequence tags that showed variation from their mature reference sequence. These variable length miRNAs are termed ‘isomirs’. Figure 2 shows an example of a mature miRNA that differs at both the 5′ and 3′ ends. For the known miRNAs sequenced in the melanoma libraries, 3′ heterogeneity occurred more commonly than 5′ heterogeneity. This is in keeping with findings from other miRNA deep sequencing studies (e.g. [24] and references therein).

Of the 847 human miRNAs annotated, we found a total of 220 hairpin regions of the pre-miRNA that were present in at least one of the libraries (Table S6). Five of these had relatively high expression levels (hsa-mir-320a, hsa-mir-1826, hsa-mir-1307, hsa-mir-29a and hsa-mir-423).

### Novel miRNAs

A total of 722 novel miRNA candidates were identified in a step-wise fashion using miRanalyzer [20] (Table S7). Firstly all of the unique sequence counts were aligned to the known pre-miRNA sequences (miRBase v12, September 2008). These sequences were then removed from the pool. The remainder were aligned to the hg18 transcriptome. The sequences that aligned to the transcriptome were also removed from the pool. All sequence reads that passed this filtering process were then mapped to the human genome. If the surrounding sequence was able to form a hairpin structure using the Vienna RNAfold package [22], indicative of a pre-miRNA, then the unique sequence was deemed to be a novel candidate miRNA. These predicted novel miRNA candidates were filtered further using CID-miRNA [21]. This reduced the list to 389 novel candidate miRNAs. RNAfold uses thermodynamic energy criteria to predict a particular RNA secondary structure based on a minimum free energy cut-off derived from thousands of experimentally measured thermodynamic parameters. The CID-miRNA program on the other hand uses a probabilistic approach to ‘learn’ model parameters from known miRNA datasets in an automated fashion. The model is composed of grammar rules that describe how a given sequence could be folded into a pre-miRNA stem loop structure. A pre-miRNA secondary structure is made of ‘stem’ interrupted with ‘symmetric’ and ‘asymmetric bulges’ and a ‘loop’ at the end. The stochastic context free grammar (SCFG) rules were designed for each of those parts of the pre-miRNA secondary structure. Each grammar rule has a probability associated with it for generating a subsequence with potential to make part of a secondary structure. A set of rules with maximum probability are used iteratively to generate a potential pre-miRNA sequence. Further filtering consisted of removing all mature candidates that were most likely rRNA degradation products and those that were found to lie in exons (>2 bp overhang into an exon). This reduced the list to 279 which have a high likelihood of being bona fide miRNAs. Table S7 shows all candidate predictions including those that have been filtered out. The table indicates which candidates have been assigned an official mirBase name. Each candidate that was subsequently named had to meet strict new criteria, specifically the abundance had to be >5 reads. Table 2 shows the total number of unique and common novel candidate miRNAs in each small RNA library. The relative abundance of these in each of the libraries is given in Table S7. The current version of miRBase (miRBase v13, March 2009) does not list any of the novel human miRNAs found in our pigment cell and melanoma libraries. In an effort to gain more confidence in the prediction of the novel pre-miRNAs, we chose a candidate from the high confidence list (MELmiRNA_677) that was selected as it had sequence counts in all libraries. A custom Taqman assay (Applied Biosystems) confirmed expression of MELmiRNA_677, giving a Pearson's correlation of 0.8 between the ΔCt value and the relative proportion of sequence counts in each library (data not shown).

**Table 2:** Total number of novel candidate miRNAs unique and common to each library.

	QF1160MB	MELB	MM653	D20	MM386	MM426	MM466	MM603	MM472	D10	D11	MEL202
Unique to a library	33	0	17	21	26	16	10	20	11	4	6	9
Present in 2 or more libraries	52	16	33	51	64	39	45	40	33	22	27	25
Totals	85	16	50	72	90	55	55	60	44	26	33	34

**Figure 3:**
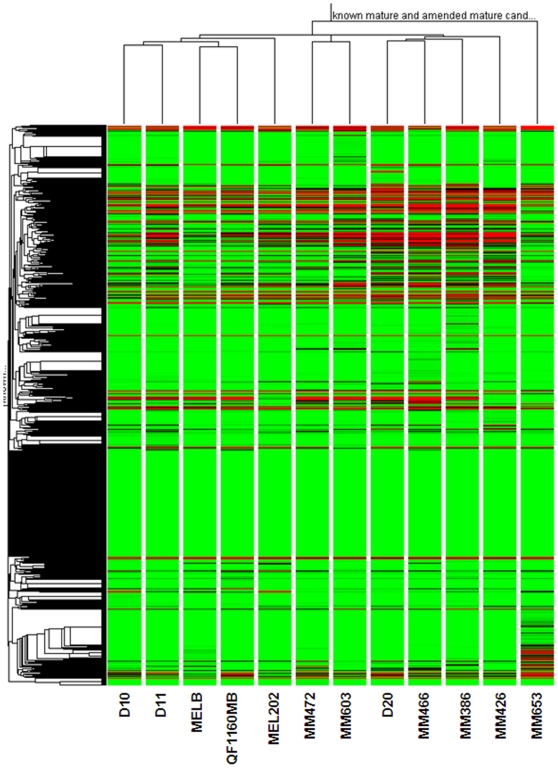
Unsupervised hierarchical clustering using the Pearson's correlation between all expressed miRNAs. QF1160MB, melanoblasts; MELB, melanocytes; D10, acral melanoma; D11, mucosal melanoma; MEL202, uveal melanoma.

Using the relative proportion of the total unique read counts against total number of reads, a condition tree (Genespring 7.3.1, Agilent) of all novel candidates plus known miRNAs gave good separation of the different histological subtypes of sample (Figure 3).

### Mirtrons

**Figure 4:**
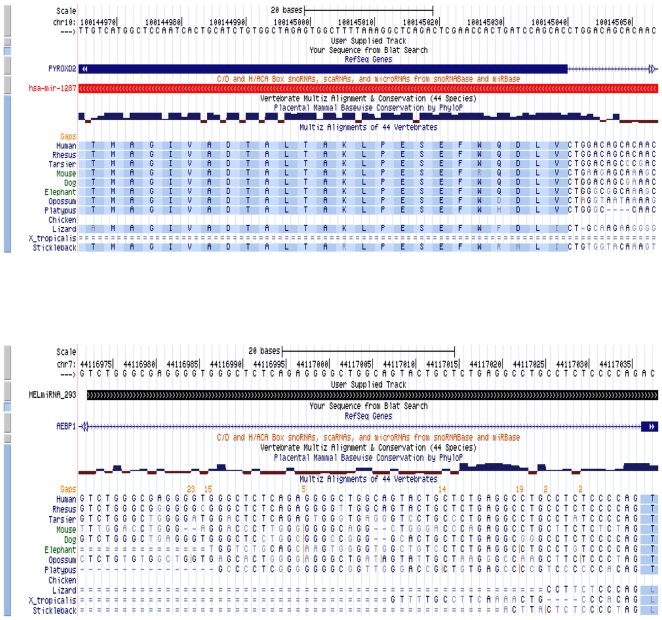
Example of sequence conservation in a known miRNA (hsa-mir-1287) and a novel candidate miRNA (MELmiRNA_293).

Mirtrons are short introns with hairpin potential that can be spliced and debranched into pre-miRNA hairpin structures, all of which appear to bypass Drosha cleavage [25]. Typically, mirtrons are anchored to splice sites both at the 5′ and 3′ ends [25]. While we did not detect any ‘typical’ mirtrons, we did find 7 known miRNAs classified as ‘atypical’ mirtrons, as described in [25] (i.e. the miRNA sequence is flanked by only one splice site), as well as 8 atypical mirtrons not previously reported (Table S8). These novel atypical mirtrons are in keeping with what is currently submitted in mirBase (miRBase v13, March 2009). However, submission of sequences to upcoming versions of mirBase require adherence to very strict criteria imposed upon high-throughput sequencing data. Some of which include the exclusion of all mature candidates that are 16 nt in length and those that are found in exons with greater than 2 bp overhang. All of the novel atypical mirtrons were observed to have some degree of cross-species sequence conservation. Figure 4 shows an example of sequence conservation in a known atypical mirtron miRNA (hsa-mir-1287) and a novel candidate atypical mirtron miRNA (MELmiRNA_293).

### Location of other miRs

Aside from the 8 novel atypical mirtrons, 196 others were found more deeply embedded within an intron, 20 were found within an exon, 17 were in a gene promoter, while the remainder were intergenic (see Table S9 for details). The genomic mapping of the known miRNAs can be found in Table S10. Of interest, some of the miRs are found within genes of particular relevance to melanoma biology, for example: *TRPM1* [26], [27].

## Discussion

The sequenced libraries were selectively chosen as they represented an array of different melanocytic subtypes ranging from normal pigment cells (melanoblasts and melanocytes) to non-UV exposed (acral, mucosal and uveal), intermittently UV-exposed and chronically UV-exposed melanomas. We also included a cell line derived from a giant congenital nevus. Figure 3 shows sample unsupervised hierarchical clustering (Pearson's correlation) based on expression of all known and predicted miRNAs. It is worthy of note that within this dendogram the group of six cutaneous melanomas cluster together.

We note that the most highly “plastic” cell type included in this study, i.e. melanoblasts, had the largest number of unique sequence tags as well as the most known and predicted miRNAs, whereas the most differentiated cell type (melanocytes) had the least (Tables S1A and S1B, and Table 2). It is thus tempting to speculate that the miRNA repertoire of a cell diminishes as it terminally differentiates. The various melanoma samples (and the nevocyte cell line) had intermediate numbers, consistent with the notion that these tumor cells have undergone some de-differentiation.

Our analyses of the sequence reads generated from the libraries was relatively stringent with the requirement of reads having to be present at least twice to be included. The removed single reads accounted for up to 20% of some libraries. While it is likely that a proportion of these single reads represent real miRNAs we set this cut-off to filter out potential sequence errors. Another stringency criterion was that each read had to map with no mismatches. Again, some of the unmatched reads may represent new miRNAs but the potential for clonal amplification of PCR-generated artefacts meant that it was more prudent for these reads to be eliminated. Additionally, as a function of depth of coverage, there may be more extremely rare miRNAs to discover in these libraries, albeit the likelihood of these having a significant impact on melanocyte biology is relatively low.

The miRanalyzer software [20] proved to be a very useful tool especially in categorising the sequence reads into different classes. This was evident in the percent of reads that mapped to the transcriptome. Due to their size it is nearly impossible to separate mature miRNA transcripts from degraded mRNAs during library preparation.

Recently, Linsen and colleagues [28] discussed the apparent sequencing bias toward certain small RNAs thereby preventing the accurate determination of their absolute numbers. Their paper used the let-7 family as an example since they account for the majority of the read counts observed. In the present study we have also noted that let-7 family members were highly expressed across all libraries and conclude that this is indeed a true reflection of their relative abundance as the mRNA expression levels of some of their known gene targets, the RAS family [29] and HMGA2 [30], show an inverse correlation with read proportion and relative gene expression (data not shown). A possible explanation for the very high read count of the let-7s, in particular let-7a and let-7f, may be because they are the master regulators of many genes involved in cell proliferation [29] and as such they require an overall high level of expression to exert an effect on each of their target genes.

The massively parallel sequencing of a diverse set of melanoma and pigment cell libraries revealed a total of 539 known mature and mature-star sequences along with the prediction of 279 candidate novel miRNAs, of which 109 were common to 2 or more libraries, with 3 present in all libraries. This is a large increase upon the number of published miRNAs currently in the miRBase database. As a proof of principle we designed a custom Taqman assay for one of the 3 miRNAs from the high confidence list (MELmiRNA_677) that was present in all libraries. We were able to confirm expression of MELmiRNA_677, which showed strong correlation between relative sequence abundance and ΔCt value (R^2^ = 0.8). These data suggest that the prediction (miRanalyzer) and filtering tools (CID-miRNA) we have employed can yield high quality predictions.

Table S7 highlights that many of the novel candidates are of quite high abundance in the libraries sequenced. This raises the question of why these novel candidates haven't been documented before? The answer may in part be due to some of these miRNAs being specific for the melanocytic lineage, or that many low abundance miRNAs are only likely to be detected through next-generation sequencing efforts. Screening of a large panel of samples from different cell types would be useful to assess these possibilities. Pigment cell specific miRNAs might be of benefit for melanoma diagnosis and early detection of distant metastases and disease recurrence by measuring the circulating levels of these potential biomarkers in blood (reviewed in [31]). The primary candidates we have identified so far that may satisfy this goal are MELmiRNA_197, MELmiRNA_434, and MELmiRNA_677, since they are present in all libraries. Moreover, candidate novel miRNAs which appear unique to a particular library may also be useful as histotype-specific markers, especially for identification of metastatic tumors of unknown origin. A more extensive analysis of a larger subset of samples of each melanoma histotype is required to answer this question. It would also be appropriate to compare miRNA profiles from uncultured melanomas and melanocytic nevi with those presented here for purified pigment cells cultured in vitro, since the in vivo microenvironment might alter the relative abundance of some miRNAs, and conceivably condition whether some miRNAs are expressed. However, in order to overcome the obvious problem of tissue heterogeneity in fresh tumor samples, such comparisons would require micro dissection of the tumor cells. Indeed, even within a tumor there could be sub-regional differences in miRNA profiles. Thus, to more comprehensively characterize the melanoma miRNAome, assessment of a large number of melanomas at different stages of progression is needed.

These data have also highlighted the importance of deep sequencing to discover and quantify miRNAs rather than simply relying on off-the-shelf microarrays that contain only a set of known miRNAs. While such arrays are useful screening tools, they do not allow for the discovery of novel candidates that may be specific to a particular cell lineage or series of samples. Moreover, they do not consider the expression of isomirs, some of which are more abundant than the annotated mature miRNA. Many of the miRNA microarrays constructed to date also lack the full complement of miRNA star sequences. Taken together, deep sequencing could be considered the gold standard for comprehensive analysis of the miRNAome.

## Materials and Methods

### Samples

Cell lines derived from melanoblasts (QF1160MB) and melanocytes (MELB) were derived from non-UV exposed neonatal foreskins [32]. A congenital giant nevus (MM653), an acral melanoma (D10), a mucosal melanoma (D11), a primary uveal melanoma (MEL202), a melanoma cell line derived from a metastasis of a primary tumor occurring on a chronically sun exposed body site (MM472), and various cell lines derived from melanoma metastases arising from primary tumors occurring on intermittently sun-exposed body sites (D20, MM386, MM426, MM466, and MM603) were included in the library preparation. Melanoblasts and melanocytes were established from neonatal foreskin tissue and cultured in as described [32], [33]. All tissue was taken with informed consent under a protocol approved by the Queensland Institute of Medical Research Human Research Ethics Committee (HREC), approval number H0311-084 (P726). All other cell lines were cultured as described in [34].

### Total RNA extraction from cultured cells

RNA was extracted from cultured cells using a miRNeasy Mini kit (QIAGEN) as per the manufacturer's instructions. This kit combines phenol/guanidine-based lysis of samples and silica membrane–based purification of total RNA which ensures no impurities remain in the sample.

### Library Construction

The small RNA libraries were constructed using a Digital Gene Expression (DGE) Small RNA sample prep kit (Illumina, San Diego, Ca) as per the manufacturer's instructions. Briefly, small RNA was gel purified from 10 µg of total RNA, 5′ and 3′ adaptors were ligated, followed by reverse transcription and library enrichment by PCR amplification. Following enrichment, the cDNA library was purified and validated for size, quality and concentration using an Agilent Bioanalyzer 2100 (Agilent Technologies, Santa Clara CA).

### Cluster Generation and SBS sequencing

Each small RNA cDNA library was denatured with 2 N NaOH to a final concentration of 0.5 nM. This was achieved by adding 1 µl of a 10 nM stock library along with 1 µl of 2 N NaOH and 18 µl of EB (QIAGEN). The denatured libraries were then diluted in hybridization buffer to the desired pM concentration. Using an Illumina Cluster Station, the diluted libraries were hybridized to an 8-lane flow cell, followed by cluster generation by isothermal amplification using a DGE-Small RNA Cluster Generation Kit v1.0 (Illumina, San Diego, CA). The prepared flowcell was then subjected to sequencing by synthesis (version 1.0) using a Genome Analyzer II, according to the manufacturer's instructions.

### Data Analysis

Following the completed sequencing runs the images were analysed using the Illumina Pipeline v1.3.2 software suite (GOAT). Firstly the images were analysed using Firecrest, which identifies clusters based on morphological features on the image and sharpens them through image filtering, removes background noise and extracts their intensities. Following image analysis bases were called using the program Bustard, which deconvolutes the signal from the clusters and applies correction for cross-talk, phasing, and prephasing (see Illumina Pipeline v1.3.2 software manual for detail). Sequence reads were then aligned to the hg18 genome using the program Eland and analysed for miRNA content using the miRanalyzer web server tool (http://web.bioinformatics.cicbiogune.es/miRNA) [20]. All data is MIAME compliant and has been deposited in GEO (www.ncbi.nlm.nih.gov/geo) (accession number GSE18381). Briefly, a perl script scans through all of the sequence outputs and assigns a copy number for each unique read. This simple input file (GSE18381) is then submitted to the webserver where it records matches to miRNAs annotated in miRBase [23], as well as perfect matches to the transcriptome. The algorithm also predicts any novel miRNAs that are present within the sequence input. In order to obtain robust data we limited the unique sequence reads fed into this algorithm to those that occurred 2 or more times. Perfect alignment with no mismatches were also part of the criteria. There is no need to remove any partial adaptor sequence from the sequence reads as the algorithm automatically trims this before alignment. The predicted novel miRNA candidates were filtered further by using CID-miRNA [21], a tool for identification of miRNA precursors in a given DNA sequence (http://mirna.jnu.ac.in/cidmirna). This algorithm utilises a probabilistic model based on stochastic context free grammar and secondary structure-based filtering systems (SCFG). CID-miRNA can be used as a command-line tool or through a web interface to analyse genomic sequences for the presence of possible pre-miRNA regions in the genome. This secondary analysis of the miRanalyzer predictions allowed for more confidence in the assignment of candidate pre-miRNAs.

The known and predicted novel candidate miRNAs were mapped to the genome following the miRanalyzer and CID-miRNA analysis. This analysis was performed to detect the presence of mirtrons. We developed a perl based pipeline to map the miRNA tags on to the known gene structures in the human genome. We used the hg18 version of human genome to extract the latest annotation. The genome coordinates for the miR-tags were obtained using miRanalyzer [20]. The transcript and coding region start and end coordinates from the hg18 annotation were used to determine the 5′UTR and 3′UTRs on the fly. The exon and intron coordinates were used as indicated in the annotation. Non-coding genes were also considered while mapping the miR-tags to the genome. A source code for this pipeline can be obtained by writing to the authors.

### Real-Time PCR confirmation

Novel predicted miRNAs were confirmed via a custom small RNA Taqman assay (Applied Biosystems). Firstly 10 ng of total RNA were reverse transcribed into cDNA using the miRNA-specific primer. From the same total RNA sample an endogenous control gene (*RNU48*) was also assayed. qRT-PCR was then performed in triplicate using a Rotorgene 6000 (QIAGEN) with the PCR products being amplified from the cDNA samples using the Taqman miRNA assay together with the Taqman Universal PCR Master Mix (Applied Biosystems). The ΔΔCT method was used to analyze the expression values.

## Supporting Information

Table S1 MiRanalyzer output summaries from each small RNA library.
(0.47 MB DOC)

Table S2 Known Melanoma mature and mature star miRNA combined.
(0.21 MB XLS)

Table S3 Common Known Mature and mature-star.
(0.05 MB XLS)

Table S4 Known miRNAs unique to each library.
(0.04 MB XLS)

Table S5 Relative ratios of miRNA compared to miRNA-star.
(0.12 MB XLS)

Table S6 Hairpins from known pre-mirs.
(0.09 MB XLS)

Table S7 Novel Melanoma miRNAs.
(0.39 MB XLS)

Table S8 Known and Novel Mirtrons.xls.
(0.02 MB XLS)

Table S9 Novel Melanoma miRNA mapped to the genome.
(0.09 MB XLS)

Table S10 Known miRNAs mapped to the genome.
(0.20 MB XLS)
