# Supplementary material for: Characterization of the Melanoma miRNAome by Deep Sequencing
Source: PLoS One. 2010 Mar 12;5(3):e9685. doi: 10.1371/journal.pone.0009685 (PMC2837346; doi:10.1371/journal.pone.0009685)
Supplement: Table S1 — MiRanalyzer output summaries from each small RNA library. (0.47 MB DOC) [file pone.0009685.s001.doc]

**Supplemental Table S1A**

| Analysis completed  You can bookmark [this page](http://web.bioinformatics.cicbiogune.es/microRNA/miRanalyser.php?launch=true&id=7562658)  Download all results in plain text [here](http://web.bioinformatics.cicbiogune.es/microRNA/webData/7562658/miRanalyser-7562658.zip) | | **Summary of input data** | | | --- | --- | | Name of input file: | QF1160MB_count2plus.txt | | Species and DB: | hsa (hg18) | | Number of allowed mismatches: | 0 | | Unique reads in input: | 77261 | | Number of reads in input  (sum of all read counts) | 5828262 | |
| --- | --- | --- | --- | --- | --- | --- | --- | --- | --- | --- | --- | --- | --- |

Known MicroRNA

| **Library/  Parameters** | **mature** | **ambiguous mature** | **mature-star** | **ambiguous mature-star** | **unknown mature-star** | **ambiguos unknown mature-star** | **hairpin** | **ambiguous hairpin** |
| --- | --- | --- | --- | --- | --- | --- | --- | --- |
| **total number** | 254 | 14 | 65 | 1 | 17 | 1 | 103 | 14 |
| **fraction (number) of known microRNAs** | 37.5%  (677) | --- | 38.2%  (170) | --- | 3.8%  (446) | --- | 15.2%  (678) | --- |
| **number of unique reads** | 6937 | 420 | 527 | 9 | 54 | 8 | 538 | 75 |
| **fraction of unique reads** | 9.0% | 0.544% | 0.682% | 0.012% | 0.070% | 0.010% | 0.696% | 0.097% |
| **read count** | 3827599 | 40026 | 26693 | 154 | 413 | 203 | 11648 | 6108 |
| **fraction of read count** | 65.7% | 0.687% | 0.458% | 0.003% | 0.007% | 0.003% | 0.200% | 0.105% |
| **links to detail pages** | [details](http://web.bioinformatics.cicbiogune.es/microRNA/miRanalyser.php?id=7562658&microSummary=true) | [details](http://web.bioinformatics.cicbiogune.es/microRNA/miRanalyser.php?id=7562658&microSummaryFam=true) | [details](http://web.bioinformatics.cicbiogune.es/microRNA/miRanalyser.php?id=7562658&microSummaryStar=true) | [details](http://web.bioinformatics.cicbiogune.es/microRNA/miRanalyser.php?id=7562658&microSummaryStarFam=true) | [details](http://web.bioinformatics.cicbiogune.es/microRNA/miRanalyser.php?id=7562658&microSummaryRev=true) | [details](http://web.bioinformatics.cicbiogune.es/microRNA/miRanalyser.php?id=7562658&microSummaryRevFam=true) | [details](http://web.bioinformatics.cicbiogune.es/microRNA/miRanalyser.php?id=7562658&microSummaryHairpin=true) | [details](http://web.bioinformatics.cicbiogune.es/microRNA/miRanalyser.php?id=7562658&microSummaryHairpinFam=true) |

Alignment to transcriptome

| **Library/Parameters** | **Transcriptome** | **Rfam** | **RepBase** | **RepeatMasker (genomic)** |
| --- | --- | --- | --- | --- |
| **number of unique reads** | 24622 | 313 | 1904 | 12394 |
| **fraction of unique reads** | 31.869% | 0.405% | 2.464% | 16.042% |
| **read count** | 857544 | 3766 | 61533 | 586134 |
| **fraction of read count** | 14.714% | 0.065% | 1.056% | 10.057% |
| **links** | [details](http://web.bioinformatics.cicbiogune.es/microRNA/miRanalyser.php?id=7562658&microSummaryMRNA=true) | [details](http://web.bioinformatics.cicbiogune.es/microRNA/miRanalyser.php?id=7562658&microSummaryRfam=true) | [details](http://web.bioinformatics.cicbiogune.es/microRNA/miRanalyser.php?id=7562658&microSummaryRepBase=true) | [details](http://web.bioinformatics.cicbiogune.es/microRNA/miRanalyser.php?id=7562658&microSummaryRM=true) |

**Predicted candidate microRNAs**

Number of predicted new microRNA: 281 (out of 285 predicted precursors )

in 572 of 77261 input reads (0.740 percent) the sequence were found to be part of a putative new microRNA which corresponds to 30634 expressed sequence reads out of 5828262 (0.526 percent)

[See detailed analysis](http://web.bioinformatics.cicbiogune.es/microRNA/miRanalyser.php?id=7562658&microSummaryCand=true)

Unmatched reads

| **Parameters** | **Filtered Reads** | **Unmapped Reads** |
| --- | --- | --- |
| **amount of unique reads** | 206 | 26007 |
| **fraction of unique reads** | 0.267% | 33.661% |
| **read count** | 1712 | 771286 |
| **fraction of read count** | 0.029% | 13.234% |
| **links** | [details](http://web.bioinformatics.cicbiogune.es/microRNA/miRanalyser.php?id=7562658&filteredReads=true) | [details](http://web.bioinformatics.cicbiogune.es/microRNA/miRanalyser.php?id=7562658&unmappedReads=true) |

Supplemental Table S1B

| Analysis completed  You can bookmark [this page](http://web.bioinformatics.cicbiogune.es/microRNA/miRanalyser.php?launch=true&id=2212652)  Download all results in plain text [here](http://web.bioinformatics.cicbiogune.es/microRNA/webData/2212652/miRanalyser-2212652.zip) | | **Summary of input data** | | | --- | --- | | Name of input file: | MELB_count2plus.txt | | Species and DB: | hsa (hg18) | | Number of allowed mismatches: | 0 | | Unique reads in input: | 17809 | | Number of reads in input  (sum of all read counts) | 1829940 | |
| --- | --- | --- | --- | --- | --- | --- | --- | --- | --- | --- | --- | --- | --- |

Known MicroRNA

| **Library/  Parameters** | **mature** | **ambiguous mature** | **mature-star** | **ambiguous mature-star** | **unknown mature-star** | **ambiguos unknown mature-star** | **hairpin** | **ambiguous hairpin** |
| --- | --- | --- | --- | --- | --- | --- | --- | --- |
| **total number** | 233 | 12 | 56 | 1 | 7 | 1 | 66 | 10 |
| **fraction (number) of known microRNAs** | 34.4%  (677) | --- | 32.9%  (170) | --- | 1.6%  (446) | --- | 9.7%  (678) | --- |
| **number of unique reads** | 4505 | 327 | 238 | 4 | 21 | 7 | 197 | 59 |
| **fraction of unique reads** | 25.3% | 1.836% | 1.336% | 0.022% | 0.118% | 0.039% | 1.106% | 0.331% |
| **read count** | 1396832 | 15715 | 5014 | 21 | 143 | 42 | 2145 | 3905 |
| **fraction of read count** | 76.3% | 0.859% | 0.274% | 0.001% | 0.008% | 0.002% | 0.117% | 0.213% |
| **links to detail pages** | [details](http://web.bioinformatics.cicbiogune.es/microRNA/miRanalyser.php?id=2212652&microSummary=true) | [details](http://web.bioinformatics.cicbiogune.es/microRNA/miRanalyser.php?id=2212652&microSummaryFam=true) | [details](http://web.bioinformatics.cicbiogune.es/microRNA/miRanalyser.php?id=2212652&microSummaryStar=true) | [details](http://web.bioinformatics.cicbiogune.es/microRNA/miRanalyser.php?id=2212652&microSummaryStarFam=true) | [details](http://web.bioinformatics.cicbiogune.es/microRNA/miRanalyser.php?id=2212652&microSummaryRev=true) | [details](http://web.bioinformatics.cicbiogune.es/microRNA/miRanalyser.php?id=2212652&microSummaryRevFam=true) | [details](http://web.bioinformatics.cicbiogune.es/microRNA/miRanalyser.php?id=2212652&microSummaryHairpin=true) | [details](http://web.bioinformatics.cicbiogune.es/microRNA/miRanalyser.php?id=2212652&microSummaryHairpinFam=true) |

Alignment to transcriptome

| **Library/Parameters** | **Transcriptome** | **Rfam** | **RepBase** | **RepeatMasker (genomic)** |
| --- | --- | --- | --- | --- |
| **number of unique reads** | 1794 | 28 | 368 | 2156 |
| **fraction of unique reads** | 10.074% | 0.157% | 2.066% | 12.106% |
| **read count** | 28651 | 154 | 10481 | 48603 |
| **fraction of read count** | 1.566% | 0.008% | 0.573% | 2.656% |
| **links** | [details](http://web.bioinformatics.cicbiogune.es/microRNA/miRanalyser.php?id=2212652&microSummaryMRNA=true) | [details](http://web.bioinformatics.cicbiogune.es/microRNA/miRanalyser.php?id=2212652&microSummaryRfam=true) | [details](http://web.bioinformatics.cicbiogune.es/microRNA/miRanalyser.php?id=2212652&microSummaryRepBase=true) | [details](http://web.bioinformatics.cicbiogune.es/microRNA/miRanalyser.php?id=2212652&microSummaryRM=true) |

**Predicted candidate microRNAs**

Number of predicted new microRNA: 53 (out of 53 predicted precursors )

in 77 of 17809 input reads (0.432 percent) the sequence were found to be part of a putative new microRNA which corresponds to 895 expressed sequence reads out of 1829940 (0.049 percent)

[See detailed analysis](http://web.bioinformatics.cicbiogune.es/microRNA/miRanalyser.php?id=2212652&microSummaryCand=true)

Unmatched reads

| **Parameters** | **Filtered Reads** | **Unmapped Reads** |
| --- | --- | --- |
| **amount of unique reads** | 30 | 4966 |
| **fraction of unique reads** | 0.168% | 27.885% |
| **read count** | 170 | 274900 |
| **fraction of read count** | 0.009% | 15.022% |
| **links** | [details](http://web.bioinformatics.cicbiogune.es/microRNA/miRanalyser.php?id=2212652&filteredReads=true) | [details](http://web.bioinformatics.cicbiogune.es/microRNA/miRanalyser.php?id=2212652&unmappedReads=true) |

**Supplemental Table S1C**

| Analysis completed  You can bookmark [this page](http://web.bioinformatics.cicbiogune.es/microRNA/miRanalyser.php?launch=true&id=680747)  Download all results in plain text [here](http://web.bioinformatics.cicbiogune.es/microRNA/webData/680747/miRanalyser-680747.zip) | | **Summary of input data** | | | --- | --- | | Name of input file: | MM653_count2plus.txt | | Species and DB: | hsa (hg18) | | Number of allowed mismatches: | 0 | | Unique reads in input: | 34903 | | Number of reads in input  (sum of all read counts) | 5131834 | |
| --- | --- | --- | --- | --- | --- | --- | --- | --- | --- | --- | --- | --- | --- |

Known MicroRNA

| **Library/  Parameters** | **mature** | **ambiguous mature** | **mature-star** | **ambiguous mature-star** | **unknown mature-star** | **ambiguos unknown mature-star** | **hairpin** | **ambiguous hairpin** |
| --- | --- | --- | --- | --- | --- | --- | --- | --- |
| **total number** | 289 | 11 | 77 | 1 | 20 | 0 | 122 | 15 |
| **fraction (number) of known microRNAs** | 42.7%  (677) | --- | 45.3%  (170) | --- | 4.5%  (446) | --- | 18.0%  (678) | --- |
| **number of unique reads** | 7128 | 678 | 612 | 3 | 76 | 0 | 582 | 163 |
| **fraction of unique reads** | 20.4% | 1.943% | 1.753% | 0.009% | 0.218% | 0.000% | 1.667% | 0.467% |
| **read count** | 3791137 | 237398 | 31826 | 46 | 2516 | 0 | 14415 | 44240 |
| **fraction of read count** | 73.9% | 4.626% | 0.620% | 0.001% | 0.049% | 0.000% | 0.281% | 0.862% |
| **links to detail pages** | [details](http://web.bioinformatics.cicbiogune.es/microRNA/miRanalyser.php?id=680747&microSummary=true) | [details](http://web.bioinformatics.cicbiogune.es/microRNA/miRanalyser.php?id=680747&microSummaryFam=true) | [details](http://web.bioinformatics.cicbiogune.es/microRNA/miRanalyser.php?id=680747&microSummaryStar=true) | [details](http://web.bioinformatics.cicbiogune.es/microRNA/miRanalyser.php?id=680747&microSummaryStarFam=true) | [details](http://web.bioinformatics.cicbiogune.es/microRNA/miRanalyser.php?id=680747&microSummaryRev=true) | no results | [details](http://web.bioinformatics.cicbiogune.es/microRNA/miRanalyser.php?id=680747&microSummaryHairpin=true) | [details](http://web.bioinformatics.cicbiogune.es/microRNA/miRanalyser.php?id=680747&microSummaryHairpinFam=true) |

Alignment to transcriptome

| **Library/Parameters** | **Transcriptome** | **Rfam** | **RepBase** | **RepeatMasker (genomic)** |
| --- | --- | --- | --- | --- |
| **number of unique reads** | 3612 | 66 | 822 | 4266 |
| **fraction of unique reads** | 10.349% | 0.189% | 2.355% | 12.222% |
| **read count** | 80626 | 506 | 42325 | 161193 |
| **fraction of read count** | 1.571% | 0.010% | 0.825% | 3.141% |
| **links** | [details](http://web.bioinformatics.cicbiogune.es/microRNA/miRanalyser.php?id=680747&microSummaryMRNA=true) | [details](http://web.bioinformatics.cicbiogune.es/microRNA/miRanalyser.php?id=680747&microSummaryRfam=true) | [details](http://web.bioinformatics.cicbiogune.es/microRNA/miRanalyser.php?id=680747&microSummaryRepBase=true) | [details](http://web.bioinformatics.cicbiogune.es/microRNA/miRanalyser.php?id=680747&microSummaryRM=true) |

**Predicted candidate microRNAs**

Number of predicted new microRNA: 115 (out of 118 predicted precursors )

in 213 of 34903 input reads (0.610 percent) the sequence were found to be part of a putative new microRNA which corresponds to 3191 expressed sequence reads out of 5131834 (0.062 percent)

[See detailed analysis](http://web.bioinformatics.cicbiogune.es/microRNA/miRanalyser.php?id=680747&microSummaryCand=true)

Unmatched reads

| **Parameters** | **Filtered Reads** | **Unmapped Reads** |
| --- | --- | --- |
| **amount of unique reads** | 937 | 10895 |
| **fraction of unique reads** | 2.685% | 31.215% |
| **read count** | 53299 | 539525 |
| **fraction of read count** | 1.039% | 10.513% |
| **links** | [details](http://web.bioinformatics.cicbiogune.es/microRNA/miRanalyser.php?id=680747&filteredReads=true) | [details](http://web.bioinformatics.cicbiogune.es/microRNA/miRanalyser.php?id=680747&unmappedReads=true) |

**Supplemental Table S1D**

| Analysis completed  You can bookmark [this page](http://web.bioinformatics.cicbiogune.es/microRNA/miRanalyser.php?launch=true&id=4894890)  Download all results in plain text [here](http://web.bioinformatics.cicbiogune.es/microRNA/webData/4894890/miRanalyser-4894890.zip) | | **Summary of input data** | | | --- | --- | | Name of input file: | D20_count2plus.txt | | Species and DB: | hsa (hg18) | | Number of allowed mismatches: | 0 | | Unique reads in input: | 52111 | | Number of reads in input  (sum of all read counts) | 4650169 | |
| --- | --- | --- | --- | --- | --- | --- | --- | --- | --- | --- | --- | --- | --- |

Known MicroRNA

| **Library/  Parameters** | **mature** | **ambiguous mature** | **mature-star** | **ambiguous mature-star** | **unknown mature-star** | **ambiguos unknown mature-star** | **hairpin** | **ambiguous hairpin** |
| --- | --- | --- | --- | --- | --- | --- | --- | --- |
| **total number** | 294 | 14 | 80 | 1 | 22 | 2 | 132 | 17 |
| **fraction (number) of known microRNAs** | 43.4%  (677) | --- | 47.1%  (170) | --- | 4.9%  (446) | --- | 19.5%  (678) | --- |
| **number of unique reads** | 7082 | 467 | 543 | 24 | 52 | 13 | 563 | 86 |
| **fraction of unique reads** | 13.6% | 0.896% | 1.042% | 0.046% | 0.100% | 0.025% | 1.080% | 0.165% |
| **read count** | 2199476 | 35703 | 17186 | 183 | 237 | 232 | 11059 | 3228 |
| **fraction of read count** | 47.3% | 0.768% | 0.370% | 0.004% | 0.005% | 0.005% | 0.238% | 0.069% |
| **links to detail pages** | [details](http://web.bioinformatics.cicbiogune.es/microRNA/miRanalyser.php?id=4894890&microSummary=true) | [details](http://web.bioinformatics.cicbiogune.es/microRNA/miRanalyser.php?id=4894890&microSummaryFam=true) | [details](http://web.bioinformatics.cicbiogune.es/microRNA/miRanalyser.php?id=4894890&microSummaryStar=true) | [details](http://web.bioinformatics.cicbiogune.es/microRNA/miRanalyser.php?id=4894890&microSummaryStarFam=true) | [details](http://web.bioinformatics.cicbiogune.es/microRNA/miRanalyser.php?id=4894890&microSummaryRev=true) | [details](http://web.bioinformatics.cicbiogune.es/microRNA/miRanalyser.php?id=4894890&microSummaryRevFam=true) | [details](http://web.bioinformatics.cicbiogune.es/microRNA/miRanalyser.php?id=4894890&microSummaryHairpin=true) | [details](http://web.bioinformatics.cicbiogune.es/microRNA/miRanalyser.php?id=4894890&microSummaryHairpinFam=true) |

Alignment to transcriptome

| **Library/Parameters** | **Transcriptome** | **Rfam** | **RepBase** | **RepeatMasker (genomic)** |
| --- | --- | --- | --- | --- |
| **number of unique reads** | 4816 | 119 | 1664 | 7139 |
| **fraction of unique reads** | 9.242% | 0.228% | 3.193% | 13.700% |
| **read count** | 146673 | 1654 | 91168 | 326502 |
| **fraction of read count** | 3.154% | 0.036% | 1.961% | 7.021% |
| **links** | [details](http://web.bioinformatics.cicbiogune.es/microRNA/miRanalyser.php?id=4894890&microSummaryMRNA=true) | [details](http://web.bioinformatics.cicbiogune.es/microRNA/miRanalyser.php?id=4894890&microSummaryRfam=true) | [details](http://web.bioinformatics.cicbiogune.es/microRNA/miRanalyser.php?id=4894890&microSummaryRepBase=true) | [details](http://web.bioinformatics.cicbiogune.es/microRNA/miRanalyser.php?id=4894890&microSummaryRM=true) |

**Predicted candidate microRNAs**

Number of predicted new microRNA: 176 (out of 181 predicted precursors )

in 354 of 52111 input reads (0.679 percent) the sequence were found to be part of a putative new microRNA which corresponds to 9748 expressed sequence reads out of 4650169 (0.210 percent)

[See detailed analysis](http://web.bioinformatics.cicbiogune.es/microRNA/miRanalyser.php?id=4894890&microSummaryCand=true)

Unmatched reads

| **Parameters** | **Filtered Reads** | **Unmapped Reads** |
| --- | --- | --- |
| **amount of unique reads** | 16 | 19951 |
| **fraction of unique reads** | 0.031% | 38.286% |
| **read count** | 45 | 1653502 |
| **fraction of read count** | 0.001% | 35.558% |
| **links** | [details](http://web.bioinformatics.cicbiogune.es/microRNA/miRanalyser.php?id=4894890&filteredReads=true) | [details](http://web.bioinformatics.cicbiogune.es/microRNA/miRanalyser.php?id=4894890&unmappedReads=true) |

**Supplemental Table S1E**

| Analysis completed  You can bookmark [this page](http://web.bioinformatics.cicbiogune.es/microRNA/miRanalyser.php?launch=true&id=3684522)  Download all results in plain text [here](http://web.bioinformatics.cicbiogune.es/microRNA/webData/3684522/miRanalyser-3684522.zip) | | **Summary of input data** | | | --- | --- | | Name of input file: | MM386_count2plus.txt | | Species and DB: | hsa (hg18) | | Number of allowed mismatches: | 0 | | Unique reads in input: | 44332 | | Number of reads in input  (sum of all read counts) | 3212548 | |
| --- | --- | --- | --- | --- | --- | --- | --- | --- | --- | --- | --- | --- | --- |

Known MicroRNA

| **Library/  Parameters** | **mature** | **ambiguous mature** | **mature-star** | **ambiguous mature-star** | **unknown mature-star** | **ambiguos unknown mature-star** | **hairpin** | **ambiguous hairpin** |
| --- | --- | --- | --- | --- | --- | --- | --- | --- |
| **total number** | 296 | 15 | 75 | 1 | 19 | 1 | 120 | 12 |
| **fraction (number) of known microRNAs** | 43.7%  (677) | --- | 44.1%  (170) | --- | 4.3%  (446) | --- | 17.7%  (678) | --- |
| **number of unique reads** | 6955 | 347 | 565 | 22 | 49 | 5 | 506 | 67 |
| **fraction of unique reads** | 15.7% | 0.783% | 1.274% | 0.050% | 0.111% | 0.011% | 1.141% | 0.151% |
| **read count** | 1582771 | 15981 | 21301 | 211 | 355 | 74 | 12368 | 4886 |
| **fraction of read count** | 49.3% | 0.497% | 0.663% | 0.007% | 0.011% | 0.002% | 0.385% | 0.152% |
| **links to detail pages** | [details](http://web.bioinformatics.cicbiogune.es/microRNA/miRanalyser.php?id=3684522&microSummary=true) | [details](http://web.bioinformatics.cicbiogune.es/microRNA/miRanalyser.php?id=3684522&microSummaryFam=true) | [details](http://web.bioinformatics.cicbiogune.es/microRNA/miRanalyser.php?id=3684522&microSummaryStar=true) | [details](http://web.bioinformatics.cicbiogune.es/microRNA/miRanalyser.php?id=3684522&microSummaryStarFam=true) | [details](http://web.bioinformatics.cicbiogune.es/microRNA/miRanalyser.php?id=3684522&microSummaryRev=true) | [details](http://web.bioinformatics.cicbiogune.es/microRNA/miRanalyser.php?id=3684522&microSummaryRevFam=true) | [details](http://web.bioinformatics.cicbiogune.es/microRNA/miRanalyser.php?id=3684522&microSummaryHairpin=true) | [details](http://web.bioinformatics.cicbiogune.es/microRNA/miRanalyser.php?id=3684522&microSummaryHairpinFam=true) |

Alignment to transcriptome

| **Library/Parameters** | **Transcriptome** | **Rfam** | **RepBase** | **RepeatMasker (genomic)** |
| --- | --- | --- | --- | --- |
| **number of unique reads** | 5938 | 165 | 1031 | 5633 |
| **fraction of unique reads** | 13.394% | 0.372% | 2.326% | 12.706% |
| **read count** | 176574 | 1633 | 34115 | 178104 |
| **fraction of read count** | 5.496% | 0.051% | 1.062% | 5.544% |
| **links** | [details](http://web.bioinformatics.cicbiogune.es/microRNA/miRanalyser.php?id=3684522&microSummaryMRNA=true) | [details](http://web.bioinformatics.cicbiogune.es/microRNA/miRanalyser.php?id=3684522&microSummaryRfam=true) | [details](http://web.bioinformatics.cicbiogune.es/microRNA/miRanalyser.php?id=3684522&microSummaryRepBase=true) | [details](http://web.bioinformatics.cicbiogune.es/microRNA/miRanalyser.php?id=3684522&microSummaryRM=true) |

**Predicted candidate microRNAs**

Number of predicted new microRNA: 199 (out of 205 predicted precursors )

in 342 of 44332 input reads (0.771 percent) the sequence were found to be part of a putative new microRNA which corresponds to 3954 expressed sequence reads out of 3212548 (0.123 percent)

[See detailed analysis](http://web.bioinformatics.cicbiogune.es/microRNA/miRanalyser.php?id=3684522&microSummaryCand=true)

Unmatched reads

| **Parameters** | **Filtered Reads** | **Unmapped Reads** |
| --- | --- | --- |
| **amount of unique reads** | 3389 | 16946 |
| **fraction of unique reads** | 7.645% | 38.225% |
| **read count** | 35379 | 1098268 |
| **fraction of read count** | 1.101% | 34.187% |
| **links** | [details](http://web.bioinformatics.cicbiogune.es/microRNA/miRanalyser.php?id=3684522&filteredReads=true) | [details](http://web.bioinformatics.cicbiogune.es/microRNA/miRanalyser.php?id=3684522&unmappedReads=true) |

**Supplemental Table S1F**

| Analysis completed  You can bookmark [this page](http://web.bioinformatics.cicbiogune.es/microRNA/miRanalyser.php?launch=true&id=8209727)  Download all results in plain text [here](http://web.bioinformatics.cicbiogune.es/microRNA/webData/8209727/miRanalyser-8209727.zip) | | **Summary of input data** | | | --- | --- | | Name of input file: | MM426_count2plus.txt | | Species and DB: | hsa (hg18) | | Number of allowed mismatches: | 0 | | Unique reads in input: | 36387 | | Number of reads in input  (sum of all read counts) | 2564037 | |
| --- | --- | --- | --- | --- | --- | --- | --- | --- | --- | --- | --- | --- | --- |

Known MicroRNA

| **Library/  Parameters** | **mature** | **ambiguous mature** | **mature-star** | **ambiguous mature-star** | **unknown mature-star** | **ambiguos unknown mature-star** | **hairpin** | **ambiguous hairpin** |
| --- | --- | --- | --- | --- | --- | --- | --- | --- |
| **total number** | 260 | 15 | 67 | 1 | 16 | 0 | 94 | 13 |
| **fraction (number) of known microRNAs** | 38.4%  (677) | --- | 39.4%  (170) | --- | 3.6%  (446) | --- | 13.9%  (678) | --- |
| **number of unique reads** | 5259 | 327 | 469 | 13 | 43 | 0 | 404 | 38 |
| **fraction of unique reads** | 14.5% | 0.899% | 1.289% | 0.036% | 0.118% | 0.000% | 1.110% | 0.104% |
| **read count** | 1374968 | 41508 | 15666 | 69 | 284 | 0 | 6598 | 1900 |
| **fraction of read count** | 53.6% | 1.619% | 0.611% | 0.003% | 0.011% | 0.000% | 0.257% | 0.074% |
| **links to detail pages** | [details](http://web.bioinformatics.cicbiogune.es/microRNA/miRanalyser.php?id=8209727&microSummary=true) | [details](http://web.bioinformatics.cicbiogune.es/microRNA/miRanalyser.php?id=8209727&microSummaryFam=true) | [details](http://web.bioinformatics.cicbiogune.es/microRNA/miRanalyser.php?id=8209727&microSummaryStar=true) | [details](http://web.bioinformatics.cicbiogune.es/microRNA/miRanalyser.php?id=8209727&microSummaryStarFam=true) | [details](http://web.bioinformatics.cicbiogune.es/microRNA/miRanalyser.php?id=8209727&microSummaryRev=true) | no results | [details](http://web.bioinformatics.cicbiogune.es/microRNA/miRanalyser.php?id=8209727&microSummaryHairpin=true) | [details](http://web.bioinformatics.cicbiogune.es/microRNA/miRanalyser.php?id=8209727&microSummaryHairpinFam=true) |

Alignment to transcriptome

| **Library/Parameters** | **Transcriptome** | **Rfam** | **RepBase** | **RepeatMasker (genomic)** |
| --- | --- | --- | --- | --- |
| **number of unique reads** | 3566 | 64 | 991 | 4631 |
| **fraction of unique reads** | 9.800% | 0.176% | 2.724% | 12.727% |
| **read count** | 90097 | 577 | 51611 | 196889 |
| **fraction of read count** | 3.514% | 0.023% | 2.013% | 7.679% |
| **links** | [details](http://web.bioinformatics.cicbiogune.es/microRNA/miRanalyser.php?id=8209727&microSummaryMRNA=true) | [details](http://web.bioinformatics.cicbiogune.es/microRNA/miRanalyser.php?id=8209727&microSummaryRfam=true) | [details](http://web.bioinformatics.cicbiogune.es/microRNA/miRanalyser.php?id=8209727&microSummaryRepBase=true) | [details](http://web.bioinformatics.cicbiogune.es/microRNA/miRanalyser.php?id=8209727&microSummaryRM=true) |

**Predicted candidate microRNAs**

Number of predicted new microRNA: 121 (out of 121 predicted precursors )

in 186 of 36387 input reads (0.511 percent) the sequence were found to be part of a putative new microRNA which corresponds to 2805 expressed sequence reads out of 2564037 (0.109 percent)

[See detailed analysis](http://web.bioinformatics.cicbiogune.es/microRNA/miRanalyser.php?id=8209727&microSummaryCand=true)

Unmatched reads

| **Parameters** | **Filtered Reads** | **Unmapped Reads** |
| --- | --- | --- |
| **amount of unique reads** | 22 | 14315 |
| **fraction of unique reads** | 0.060% | 39.341% |
| **read count** | 74 | 689479 |
| **fraction of read count** | 0.003% | 26.890% |
| **links** | [details](http://web.bioinformatics.cicbiogune.es/microRNA/miRanalyser.php?id=8209727&filteredReads=true) | [details](http://web.bioinformatics.cicbiogune.es/microRNA/miRanalyser.php?id=8209727&unmappedReads=true) |

**Supplemental Table S1G**

| Analysis completed  You can bookmark [this page](http://web.bioinformatics.cicbiogune.es/microRNA/miRanalyser.php?launch=true&id=581916)  Download all results in plain text [here](http://web.bioinformatics.cicbiogune.es/microRNA/webData/581916/miRanalyser-581916.zip) | | **Summary of input data** | | | --- | --- | | Name of input file: | MM466_count2plus.txt | | Species and DB: | hsa (hg18) | | Number of allowed mismatches: | 0 | | Unique reads in input: | 47977 | | Number of reads in input  (sum of all read counts) | 2588736 | |
| --- | --- | --- | --- | --- | --- | --- | --- | --- | --- | --- | --- | --- | --- |

Known MicroRNA

| **Library/  Parameters** | **mature** | **ambiguous mature** | **mature-star** | **ambiguous mature-star** | **unknown mature-star** | **ambiguos unknown mature-star** | **hairpin** | **ambiguous hairpin** |
| --- | --- | --- | --- | --- | --- | --- | --- | --- |
| **total number** | 272 | 17 | 70 | 1 | 12 | 2 | 101 | 12 |
| **fraction (number) of known microRNAs** | 40.2%  (677) | --- | 41.2%  (170) | --- | 2.7%  (446) | --- | 14.9%  (678) | --- |
| **number of unique reads** | 5786 | 310 | 384 | 9 | 28 | 17 | 451 | 64 |
| **fraction of unique reads** | 12.1% | 0.646% | 0.800% | 0.019% | 0.058% | 0.035% | 0.940% | 0.133% |
| **read count** | 1164419 | 31851 | 10273 | 24 | 157 | 384 | 10634 | 3681 |
| **fraction of read count** | 45.0% | 1.230% | 0.397% | 0.001% | 0.006% | 0.015% | 0.411% | 0.142% |
| **links to detail pages** | [details](http://web.bioinformatics.cicbiogune.es/microRNA/miRanalyser.php?id=581916&microSummary=true) | [details](http://web.bioinformatics.cicbiogune.es/microRNA/miRanalyser.php?id=581916&microSummaryFam=true) | [details](http://web.bioinformatics.cicbiogune.es/microRNA/miRanalyser.php?id=581916&microSummaryStar=true) | [details](http://web.bioinformatics.cicbiogune.es/microRNA/miRanalyser.php?id=581916&microSummaryStarFam=true) | [details](http://web.bioinformatics.cicbiogune.es/microRNA/miRanalyser.php?id=581916&microSummaryRev=true) | [details](http://web.bioinformatics.cicbiogune.es/microRNA/miRanalyser.php?id=581916&microSummaryRevFam=true) | [details](http://web.bioinformatics.cicbiogune.es/microRNA/miRanalyser.php?id=581916&microSummaryHairpin=true) | [details](http://web.bioinformatics.cicbiogune.es/microRNA/miRanalyser.php?id=581916&microSummaryHairpinFam=true) |

Alignment to transcriptome

| **Library/Parameters** | **Transcriptome** | **Rfam** | **RepBase** | **RepeatMasker (genomic)** |
| --- | --- | --- | --- | --- |
| **number of unique reads** | 7119 | 52 | 1202 | 7229 |
| **fraction of unique reads** | 14.838% | 0.108% | 2.505% | 15.068% |
| **read count** | 176841 | 357 | 63033 | 267623 |
| **fraction of read count** | 6.831% | 0.014% | 2.435% | 10.338% |
| **links** | [details](http://web.bioinformatics.cicbiogune.es/microRNA/miRanalyser.php?id=581916&microSummaryMRNA=true) | [details](http://web.bioinformatics.cicbiogune.es/microRNA/miRanalyser.php?id=581916&microSummaryRfam=true) | [details](http://web.bioinformatics.cicbiogune.es/microRNA/miRanalyser.php?id=581916&microSummaryRepBase=true) | [details](http://web.bioinformatics.cicbiogune.es/microRNA/miRanalyser.php?id=581916&microSummaryRM=true) |

**Predicted candidate microRNAs**

Number of predicted new microRNA: 147 (out of 151 predicted precursors )

in 264 of 47977 input reads (0.550 percent) the sequence were found to be part of a putative new microRNA which corresponds to 5559 expressed sequence reads out of 2588736 (0.215 percent)

[See detailed analysis](http://web.bioinformatics.cicbiogune.es/microRNA/miRanalyser.php?id=581916&microSummaryCand=true)

Unmatched reads

| **Parameters** | **Filtered Reads** | **Unmapped Reads** |
| --- | --- | --- |
| **amount of unique reads** | 22 | 19033 |
| **fraction of unique reads** | 0.046% | 39.671% |
| **read count** | 67 | 803450 |
| **fraction of read count** | 0.003% | 31.036% |
| **links** | [details](http://web.bioinformatics.cicbiogune.es/microRNA/miRanalyser.php?id=581916&filteredReads=true) | [details](http://web.bioinformatics.cicbiogune.es/microRNA/miRanalyser.php?id=581916&unmappedReads=true) |

**Supplemental Table S1H**

| Analysis completed  You can bookmark [this page](http://web.bioinformatics.cicbiogune.es/microRNA/miRanalyser.php?launch=true&id=5602118)  Download all results in plain text [here](http://web.bioinformatics.cicbiogune.es/microRNA/webData/5602118/miRanalyser-5602118.zip) | | **Summary of input data** | | | --- | --- | | Name of input file: | MM603_count2plus.txt | | Species and DB: | hsa (hg18) | | Number of allowed mismatches: | 0 | | Unique reads in input: | 34844 | | Number of reads in input  (sum of all read counts) | 2657924 | |
| --- | --- | --- | --- | --- | --- | --- | --- | --- | --- | --- | --- | --- | --- |

Known MicroRNA

| **Library/  Parameters** | **mature** | **ambiguous mature** | **mature-star** | **ambiguous mature-star** | **unknown mature-star** | **ambiguos unknown mature-star** | **hairpin** | **ambiguous hairpin** |
| --- | --- | --- | --- | --- | --- | --- | --- | --- |
| **total number** | 293 | 14 | 72 | 1 | 11 | 1 | 104 | 14 |
| **fraction (number) of known microRNAs** | 43.3%  (677) | --- | 42.4%  (170) | --- | 2.5%  (446) | --- | 15.3%  (678) | --- |
| **number of unique reads** | 5064 | 263 | 349 | 3 | 19 | 6 | 341 | 53 |
| **fraction of unique reads** | 14.5% | 0.755% | 1.002% | 0.009% | 0.055% | 0.017% | 0.979% | 0.152% |
| **read count** | 1560761 | 23238 | 9463 | 7 | 81 | 93 | 5436 | 2794 |
| **fraction of read count** | 58.7% | 0.874% | 0.356% | 0.000% | 0.003% | 0.003% | 0.205% | 0.105% |
| **links to detail pages** | [details](http://web.bioinformatics.cicbiogune.es/microRNA/miRanalyser.php?id=5602118&microSummary=true) | [details](http://web.bioinformatics.cicbiogune.es/microRNA/miRanalyser.php?id=5602118&microSummaryFam=true) | [details](http://web.bioinformatics.cicbiogune.es/microRNA/miRanalyser.php?id=5602118&microSummaryStar=true) | [details](http://web.bioinformatics.cicbiogune.es/microRNA/miRanalyser.php?id=5602118&microSummaryStarFam=true) | [details](http://web.bioinformatics.cicbiogune.es/microRNA/miRanalyser.php?id=5602118&microSummaryRev=true) | [details](http://web.bioinformatics.cicbiogune.es/microRNA/miRanalyser.php?id=5602118&microSummaryRevFam=true) | [details](http://web.bioinformatics.cicbiogune.es/microRNA/miRanalyser.php?id=5602118&microSummaryHairpin=true) | [details](http://web.bioinformatics.cicbiogune.es/microRNA/miRanalyser.php?id=5602118&microSummaryHairpinFam=true) |

Alignment to transcriptome

| **Library/Parameters** | **Transcriptome** | **Rfam** | **RepBase** | **RepeatMasker (genomic)** |
| --- | --- | --- | --- | --- |
| **number of unique reads** | 2982 | 58 | 815 | 4470 |
| **fraction of unique reads** | 8.558% | 0.166% | 2.339% | 12.829% |
| **read count** | 70769 | 486 | 28365 | 149794 |
| **fraction of read count** | 2.663% | 0.018% | 1.067% | 5.636% |
| **links** | [details](http://web.bioinformatics.cicbiogune.es/microRNA/miRanalyser.php?id=5602118&microSummaryMRNA=true) | [details](http://web.bioinformatics.cicbiogune.es/microRNA/miRanalyser.php?id=5602118&microSummaryRfam=true) | [details](http://web.bioinformatics.cicbiogune.es/microRNA/miRanalyser.php?id=5602118&microSummaryRepBase=true) | [details](http://web.bioinformatics.cicbiogune.es/microRNA/miRanalyser.php?id=5602118&microSummaryRM=true) |

**Predicted candidate microRNAs**

Number of predicted new microRNA: 131 (out of 134 predicted precursors )

in 233 of 34844 input reads (0.669 percent) the sequence were found to be part of a putative new microRNA which corresponds to 3142 expressed sequence reads out of 2657924 (0.118 percent)

[See detailed analysis](http://web.bioinformatics.cicbiogune.es/microRNA/miRanalyser.php?id=5602118&microSummaryCand=true)

Unmatched reads

| **Parameters** | **Filtered Reads** | **Unmapped Reads** |
| --- | --- | --- |
| **amount of unique reads** | 20 | 14263 |
| **fraction of unique reads** | 0.057% | 40.934% |
| **read count** | 70 | 737603 |
| **fraction of read count** | 0.003% | 27.751% |
| **links** | [details](http://web.bioinformatics.cicbiogune.es/microRNA/miRanalyser.php?id=5602118&filteredReads=true) | [details](http://web.bioinformatics.cicbiogune.es/microRNA/miRanalyser.php?id=5602118&unmappedReads=true) |

**Supplemental Table S1I**

| Analysis completed  You can bookmark [this page](http://web.bioinformatics.cicbiogune.es/microRNA/miRanalyser.php?launch=true&id=9291182)  Download all results in plain text [here](http://web.bioinformatics.cicbiogune.es/microRNA/webData/9291182/miRanalyser-9291182.zip) | | **Summary of input data** | | | --- | --- | | Name of input file: | MM472_count1plus.txt | | Species and DB: | hsa (hg18) | | Number of allowed mismatches: | 0 | | Unique reads in input: | 24474 | | Number of reads in input  (sum of all read counts) | 2519368 | |
| --- | --- | --- | --- | --- | --- | --- | --- | --- | --- | --- | --- | --- | --- |

Known MicroRNA

| **Library/  Parameters** | **mature** | **ambiguous mature** | **mature-star** | **ambiguous mature-star** | **unknown mature-star** | **ambiguos unknown mature-star** | **hairpin** | **ambiguous hairpin** |
| --- | --- | --- | --- | --- | --- | --- | --- | --- |
| **total number** | 249 | 15 | 65 | 1 | 8 | 2 | 96 | 15 |
| **fraction (number) of known microRNAs** | 36.8%  (677) | --- | 38.2%  (170) | --- | 1.8%  (446) | --- | 14.2%  (678) | --- |
| **number of unique reads** | 5716 | 336 | 399 | 4 | 19 | 12 | 346 | 56 |
| **fraction of unique reads** | 23.4% | 1.373% | 1.630% | 0.016% | 0.078% | 0.049% | 1.414% | 0.229% |
| **read count** | 1842812 | 20208 | 15538 | 25 | 92 | 238 | 4704 | 2934 |
| **fraction of read count** | 73.1% | 0.802% | 0.617% | 0.001% | 0.004% | 0.009% | 0.187% | 0.116% |
| **links to detail pages** | [details](http://web.bioinformatics.cicbiogune.es/microRNA/miRanalyser.php?id=9291182&microSummary=true) | [details](http://web.bioinformatics.cicbiogune.es/microRNA/miRanalyser.php?id=9291182&microSummaryFam=true) | [details](http://web.bioinformatics.cicbiogune.es/microRNA/miRanalyser.php?id=9291182&microSummaryStar=true) | [details](http://web.bioinformatics.cicbiogune.es/microRNA/miRanalyser.php?id=9291182&microSummaryStarFam=true) | [details](http://web.bioinformatics.cicbiogune.es/microRNA/miRanalyser.php?id=9291182&microSummaryRev=true) | [details](http://web.bioinformatics.cicbiogune.es/microRNA/miRanalyser.php?id=9291182&microSummaryRevFam=true) | [details](http://web.bioinformatics.cicbiogune.es/microRNA/miRanalyser.php?id=9291182&microSummaryHairpin=true) | [details](http://web.bioinformatics.cicbiogune.es/microRNA/miRanalyser.php?id=9291182&microSummaryHairpinFam=true) |

Alignment to transcriptome

| **Library/Parameters** | **Transcriptome** | **Rfam** | **RepBase** | **RepeatMasker (genomic)** |
| --- | --- | --- | --- | --- |
| **number of unique reads** | 2483 | 39 | 746 | 3257 |
| **fraction of unique reads** | 10.145% | 0.159% | 3.048% | 13.308% |
| **read count** | 75786 | 397 | 32254 | 122105 |
| **fraction of read count** | 3.008% | 0.016% | 1.280% | 4.847% |
| **links** | [details](http://web.bioinformatics.cicbiogune.es/microRNA/miRanalyser.php?id=9291182&microSummaryMRNA=true) | [details](http://web.bioinformatics.cicbiogune.es/microRNA/miRanalyser.php?id=9291182&microSummaryRfam=true) | [details](http://web.bioinformatics.cicbiogune.es/microRNA/miRanalyser.php?id=9291182&microSummaryRepBase=true) | [details](http://web.bioinformatics.cicbiogune.es/microRNA/miRanalyser.php?id=9291182&microSummaryRM=true) |

**Predicted candidate microRNAs**

Number of predicted new microRNA: 106 (out of 109 predicted precursors )

in 167 of 24474 input reads (0.682 percent) the sequence were found to be part of a putative new microRNA which corresponds to 2302 expressed sequence reads out of 2519368 (0.091 percent)

[See detailed analysis](http://web.bioinformatics.cicbiogune.es/microRNA/miRanalyser.php?id=9291182&microSummaryCand=true)

Unmatched reads

| **Parameters** | **Filtered Reads** | **Unmapped Reads** |
| --- | --- | --- |
| **amount of unique reads** | 38 | 6754 |
| **fraction of unique reads** | 0.155% | 27.597% |
| **read count** | 289 | 365575 |
| **fraction of read count** | 0.011% | 14.511% |
| **links** | [details](http://web.bioinformatics.cicbiogune.es/microRNA/miRanalyser.php?id=9291182&filteredReads=true) | [details](http://web.bioinformatics.cicbiogune.es/microRNA/miRanalyser.php?id=9291182&unmappedReads=true) |

**Supplemental Table S1J**

| Analysis completed  You can bookmark [this page](http://web.bioinformatics.cicbiogune.es/microRNA/miRanalyser.php?launch=true&id=9080930)  Download all results in plain text [here](http://web.bioinformatics.cicbiogune.es/microRNA/webData/9080930/miRanalyser-9080930.zip) | | **Summary of input data** | | | --- | --- | | Name of input file: | D10_count1plus.txt | | Species and DB: | hsa (hg18) | | Number of allowed mismatches: | 0 | | Unique reads in input: | 17278 | | Number of reads in input  (sum of all read counts) | 1505552 | |
| --- | --- | --- | --- | --- | --- | --- | --- | --- | --- | --- | --- | --- | --- |

Known MicroRNA

| **Library/  Parameters** | **mature** | **ambiguous mature** | **mature-star** | **ambiguous mature-star** | **unknown mature-star** | **ambiguos unknown mature-star** | **hairpin** | **ambiguous hairpin** |
| --- | --- | --- | --- | --- | --- | --- | --- | --- |
| **total number** | 228 | 14 | 52 | 1 | 9 | 1 | 59 | 9 |
| **fraction (number) of known microRNAs** | 33.7%  (677) | --- | 30.6%  (170) | --- | 2.0%  (446) | --- | 8.7%  (678) | --- |
| **number of unique reads** | 3950 | 217 | 258 | 3 | 15 | 4 | 214 | 29 |
| **fraction of unique reads** | 22.9% | 1.256% | 1.493% | 0.017% | 0.087% | 0.023% | 1.239% | 0.168% |
| **read count** | 1145926 | 24200 | 13321 | 8 | 73 | 16 | 3285 | 1550 |
| **fraction of read count** | 76.1% | 1.607% | 0.885% | 0.001% | 0.005% | 0.001% | 0.218% | 0.103% |
| **links to detail pages** | [details](http://web.bioinformatics.cicbiogune.es/microRNA/miRanalyser.php?id=9080930&microSummary=true) | [details](http://web.bioinformatics.cicbiogune.es/microRNA/miRanalyser.php?id=9080930&microSummaryFam=true) | [details](http://web.bioinformatics.cicbiogune.es/microRNA/miRanalyser.php?id=9080930&microSummaryStar=true) | [details](http://web.bioinformatics.cicbiogune.es/microRNA/miRanalyser.php?id=9080930&microSummaryStarFam=true) | [details](http://web.bioinformatics.cicbiogune.es/microRNA/miRanalyser.php?id=9080930&microSummaryRev=true) | [details](http://web.bioinformatics.cicbiogune.es/microRNA/miRanalyser.php?id=9080930&microSummaryRevFam=true) | [details](http://web.bioinformatics.cicbiogune.es/microRNA/miRanalyser.php?id=9080930&microSummaryHairpin=true) | [details](http://web.bioinformatics.cicbiogune.es/microRNA/miRanalyser.php?id=9080930&microSummaryHairpinFam=true) |

Alignment to transcriptome

| **Library/Parameters** | **Transcriptome** | **Rfam** | **RepBase** | **RepeatMasker (genomic)** |
| --- | --- | --- | --- | --- |
| **number of unique reads** | 1784 | 26 | 429 | 2441 |
| **fraction of unique reads** | 10.325% | 0.150% | 2.483% | 14.128% |
| **read count** | 46899 | 188 | 15625 | 80047 |
| **fraction of read count** | 3.115% | 0.012% | 1.038% | 5.317% |
| **links** | [details](http://web.bioinformatics.cicbiogune.es/microRNA/miRanalyser.php?id=9080930&microSummaryMRNA=true) | [details](http://web.bioinformatics.cicbiogune.es/microRNA/miRanalyser.php?id=9080930&microSummaryRfam=true) | [details](http://web.bioinformatics.cicbiogune.es/microRNA/miRanalyser.php?id=9080930&microSummaryRepBase=true) | [details](http://web.bioinformatics.cicbiogune.es/microRNA/miRanalyser.php?id=9080930&microSummaryRM=true) |

**Predicted candidate microRNAs**

Number of predicted new microRNA: 54 (out of 54 predicted precursors )

in 82 of 17278 input reads (0.475 percent) the sequence were found to be part of a putative new microRNA which corresponds to 4495 expressed sequence reads out of 1505552 (0.299 percent)

[See detailed analysis](http://web.bioinformatics.cicbiogune.es/microRNA/miRanalyser.php?id=9080930&microSummaryCand=true)

Unmatched reads

| **Parameters** | **Filtered Reads** | **Unmapped Reads** |
| --- | --- | --- |
| **amount of unique reads** | 1539 | 4361 |
| **fraction of unique reads** | 8.907% | 25.240% |
| **read count** | 54630 | 111763 |
| **fraction of read count** | 3.629% | 7.423% |
| **links** | [details](http://web.bioinformatics.cicbiogune.es/microRNA/miRanalyser.php?id=9080930&filteredReads=true) | [details](http://web.bioinformatics.cicbiogune.es/microRNA/miRanalyser.php?id=9080930&unmappedReads=true) |

**Supplemental Table S1K**

| Analysis completed  You can bookmark [this page](http://web.bioinformatics.cicbiogune.es/microRNA/miRanalyser.php?launch=true&id=6459732)  Download all results in plain text [here](http://web.bioinformatics.cicbiogune.es/microRNA/webData/6459732/miRanalyser-6459732.zip) | | **Summary of input data** | | | --- | --- | | Name of input file: | D11_count1plus.txt | | Species and DB: | hsa (hg18) | | Number of allowed mismatches: | 0 | | Unique reads in input: | 20254 | | Number of reads in input  (sum of all read counts) | 1250799 | |
| --- | --- | --- | --- | --- | --- | --- | --- | --- | --- | --- | --- | --- | --- |

Known MicroRNA

| **Library/  Parameters** | **mature** | **ambiguous mature** | **mature-star** | **ambiguous mature-star** | **unknown mature-star** | **ambiguos unknown mature-star** | **hairpin** | **ambiguous hairpin** |
| --- | --- | --- | --- | --- | --- | --- | --- | --- |
| **total number** | 245 | 13 | 55 | 1 | 11 | 1 | 71 | 13 |
| **fraction (number) of known microRNAs** | 36.2%  (677) | --- | 32.4%  (170) | --- | 2.5%  (446) | --- | 10.5%  (678) | --- |
| **number of unique reads** | 4158 | 244 | 290 | 5 | 20 | 3 | 229 | 53 |
| **fraction of unique reads** | 20.5% | 1.205% | 1.432% | 0.025% | 0.099% | 0.015% | 1.131% | 0.262% |
| **read count** | 885339 | 16308 | 7540 | 22 | 156 | 13 | 2676 | 5470 |
| **fraction of read count** | 70.8% | 1.304% | 0.603% | 0.002% | 0.012% | 0.001% | 0.214% | 0.437% |
| **links to detail pages** | [details](http://web.bioinformatics.cicbiogune.es/microRNA/miRanalyser.php?id=6459732&microSummary=true) | [details](http://web.bioinformatics.cicbiogune.es/microRNA/miRanalyser.php?id=6459732&microSummaryFam=true) | [details](http://web.bioinformatics.cicbiogune.es/microRNA/miRanalyser.php?id=6459732&microSummaryStar=true) | [details](http://web.bioinformatics.cicbiogune.es/microRNA/miRanalyser.php?id=6459732&microSummaryStarFam=true) | [details](http://web.bioinformatics.cicbiogune.es/microRNA/miRanalyser.php?id=6459732&microSummaryRev=true) | [details](http://web.bioinformatics.cicbiogune.es/microRNA/miRanalyser.php?id=6459732&microSummaryRevFam=true) | [details](http://web.bioinformatics.cicbiogune.es/microRNA/miRanalyser.php?id=6459732&microSummaryHairpin=true) | [details](http://web.bioinformatics.cicbiogune.es/microRNA/miRanalyser.php?id=6459732&microSummaryHairpinFam=true) |

Alignment to transcriptome

| **Library/Parameters** | **Transcriptome** | **Rfam** | **RepBase** | **RepeatMasker (genomic)** |
| --- | --- | --- | --- | --- |
| **number of unique reads** | 2162 | 27 | 604 | 2997 |
| **fraction of unique reads** | 10.674% | 0.133% | 2.982% | 14.797% |
| **read count** | 38185 | 173 | 19533 | 87145 |
| **fraction of read count** | 3.053% | 0.014% | 1.562% | 6.967% |
| **links** | [details](http://web.bioinformatics.cicbiogune.es/microRNA/miRanalyser.php?id=6459732&microSummaryMRNA=true) | [details](http://web.bioinformatics.cicbiogune.es/microRNA/miRanalyser.php?id=6459732&microSummaryRfam=true) | [details](http://web.bioinformatics.cicbiogune.es/microRNA/miRanalyser.php?id=6459732&microSummaryRepBase=true) | [details](http://web.bioinformatics.cicbiogune.es/microRNA/miRanalyser.php?id=6459732&microSummaryRM=true) |

**Predicted candidate microRNAs**

Number of predicted new microRNA: 78 (out of 80 predicted precursors )

in 132 of 20254 input reads (0.652 percent) the sequence were found to be part of a putative new microRNA which corresponds to 3206 expressed sequence reads out of 1250799 (0.256 percent)

[See detailed analysis](http://web.bioinformatics.cicbiogune.es/microRNA/miRanalyser.php?id=6459732&microSummaryCand=true)

Unmatched reads

| **Parameters** | **Filtered Reads** | **Unmapped Reads** |
| --- | --- | --- |
| **amount of unique reads** | 29 | 6019 |
| **fraction of unique reads** | 0.143% | 29.718% |
| **read count** | 111 | 153342 |
| **fraction of read count** | 0.009% | 12.260% |
| **links** | [details](http://web.bioinformatics.cicbiogune.es/microRNA/miRanalyser.php?id=6459732&filteredReads=true) | [details](http://web.bioinformatics.cicbiogune.es/microRNA/miRanalyser.php?id=6459732&unmappedReads=true) |

**Supplemental Table S1L**

| Analysis completed  You can bookmark [this page](http://web.bioinformatics.cicbiogune.es/microRNA/miRanalyser.php?launch=true&id=2748906)  Download all results in plain text [here](http://web.bioinformatics.cicbiogune.es/microRNA/webData/2748906/miRanalyser-2748906.zip) | | **Summary of input data** | | | --- | --- | | Name of input file: | MEL202_count2plus.txt | | Species and DB: | hsa (hg18) | | Number of allowed mismatches: | 0 | | Unique reads in input: | 24254 | | Number of reads in input  (sum of all read counts) | 2005205 | |
| --- | --- | --- | --- | --- | --- | --- | --- | --- | --- | --- | --- | --- | --- |

Known MicroRNA

| **Library/  Parameters** | **mature** | **ambiguous mature** | **mature-star** | **ambiguous mature-star** | **unknown mature-star** | **ambiguos unknown mature-star** | **hairpin** | **ambiguous hairpin** |
| --- | --- | --- | --- | --- | --- | --- | --- | --- |
| **total number** | 241 | 15 | 61 | 1 | 11 | 1 | 90 | 10 |
| **fraction (number) of known microRNAs** | 35.6%  (677) | --- | 35.9%  (170) | --- | 2.5%  (446) | --- | 13.3%  (678) | --- |
| **number of unique reads** | 4940 | 356 | 342 | 4 | 26 | 1 | 298 | 66 |
| **fraction of unique reads** | 20.4% | 1.468% | 1.410% | 0.016% | 0.107% | 0.004% | 1.229% | 0.272% |
| **read count** | 1555740 | 26556 | 10296 | 24 | 120 | 2 | 4056 | 6193 |
| **fraction of read count** | 77.6% | 1.324% | 0.513% | 0.001% | 0.006% | 0.000% | 0.202% | 0.309% |
| **links to detail pages** | [details](http://web.bioinformatics.cicbiogune.es/microRNA/miRanalyser.php?id=2748906&microSummary=true) | [details](http://web.bioinformatics.cicbiogune.es/microRNA/miRanalyser.php?id=2748906&microSummaryFam=true) | [details](http://web.bioinformatics.cicbiogune.es/microRNA/miRanalyser.php?id=2748906&microSummaryStar=true) | [details](http://web.bioinformatics.cicbiogune.es/microRNA/miRanalyser.php?id=2748906&microSummaryStarFam=true) | [details](http://web.bioinformatics.cicbiogune.es/microRNA/miRanalyser.php?id=2748906&microSummaryRev=true) | [details](http://web.bioinformatics.cicbiogune.es/microRNA/miRanalyser.php?id=2748906&microSummaryRevFam=true) | [details](http://web.bioinformatics.cicbiogune.es/microRNA/miRanalyser.php?id=2748906&microSummaryHairpin=true) | [details](http://web.bioinformatics.cicbiogune.es/microRNA/miRanalyser.php?id=2748906&microSummaryHairpinFam=true) |

Alignment to transcriptome

| **Library/Parameters** | **Transcriptome** | **Rfam** | **RepBase** | **RepeatMasker (genomic)** |
| --- | --- | --- | --- | --- |
| **number of unique reads** | 2799 | 48 | 725 | 3325 |
| **fraction of unique reads** | 11.540% | 0.198% | 2.989% | 13.709% |
| **read count** | 50152 | 256 | 23015 | 103173 |
| **fraction of read count** | 2.501% | 0.013% | 1.148% | 5.145% |
| **links** | [details](http://web.bioinformatics.cicbiogune.es/microRNA/miRanalyser.php?id=2748906&microSummaryMRNA=true) | [details](http://web.bioinformatics.cicbiogune.es/microRNA/miRanalyser.php?id=2748906&microSummaryRfam=true) | [details](http://web.bioinformatics.cicbiogune.es/microRNA/miRanalyser.php?id=2748906&microSummaryRepBase=true) | [details](http://web.bioinformatics.cicbiogune.es/microRNA/miRanalyser.php?id=2748906&microSummaryRM=true) |

**Predicted candidate microRNAs**

Number of predicted new microRNA: 78 (out of 78 predicted precursors )

in 136 of 24254 input reads (0.561 percent) the sequence were found to be part of a putative new microRNA which corresponds to 6024 expressed sequence reads out of 2005205 (0.300 percent)

[See detailed analysis](http://web.bioinformatics.cicbiogune.es/microRNA/miRanalyser.php?id=2748906&microSummaryCand=true)

Unmatched reads

| **Parameters** | **Filtered Reads** | **Unmapped Reads** |
| --- | --- | --- |
| **amount of unique reads** | 30 | 6681 |
| **fraction of unique reads** | 0.124% | 27.546% |
| **read count** | 225 | 167559 |
| **fraction of read count** | 0.011% | 8.356% |
| **links** | [details](http://web.bioinformatics.cicbiogune.es/microRNA/miRanalyser.php?id=2748906&filteredReads=true) | [details](http://web.bioinformatics.cicbiogune.es/microRNA/miRanalyser.php?id=2748906&unmappedReads=true) |
